# Assessment of the Status of Water, Sanitation and Hygiene (WASH) Services at Primary Schools in uMfolozi Local Municipality, Kwa-Zulu Natal, South Africa

**DOI:** 10.3390/ijerph22030360

**Published:** 2025-02-28

**Authors:** Lindokuhle C. Radebe, Matlou I. Mokgobu, Gomotsegang F. Molelekwa, Matodzi M. Mokoena

**Affiliations:** 1 Department of Environmental Health, Tshwane University of Technology, Private Bag X680, Pretoria Campus, Pretoria 0001, South Africa; mokgobumi@tut.ac.za (M.I.M.); molelekwagf@tut.ac.za (G.F.M.); mokoenamm1@tut.ac.za (M.M.M.); 2 Regional Water and Environmental Sanitation Centre, Department of Civil Engineering, Kwame Nkrumah University of Science and Technology, Kumasi AK-039-5028, Ghana

**Keywords:** school water sanitation and hygiene, safe drinking water, water availability and reliability, handwashing, drinking points, sanitation facilities, water supply infrastructure

## Abstract

This study assessed the status of water, sanitation, and hygiene (WASH) services at (49) selected primary schools in uMfolozi Local Municipality, which is situated in the province of Kwa-Zulu Natal in South Africa. Data were collected using an observational checklist tool and by conducting a walk-through survey to inspect the conditions of sanitary facilities, observe the hand-washing practices of the school learners, and analyse the accessibility to safe drinking water in school premises. The data were analysed with the Statistical Package for Social Science Version 29. This study revealed that there is easy access to safe drinking water in all but one school. The dependability of the water supply seemed to be one of the most urgent problems in every school, even though all of them have some kind of drinking water infrastructure on their grounds. Municipal water (*n* = 25, 36%) and rainwater (*n* = 25, 36%) were the most common type of water used in schools compared to borehole (*n* = 15, 22%) and tanker truck water (*n* = 4, 6%). Schools must have a reserved water supply because of the inconsistent supply of municipal water, and because rainwater is a seasonal harvest while borehole water may be affected by factors like load-shedding. The UNICEF-described ratio of one tap or disperser per fifty learners suggests that the water taps in the schoolyard were insufficient in some schools (*n* = 25, 36%). Rainwater is collected through a gutter system in the school building roofs and stored in 5000–10,000 Jojo tanks. Borehole water is pumped into Jojo tanks at an elevated position where it is stored, and learners receive the water through taps connected to the borehole tanks. During an emergency when there is no water supply from other sources, tanker trucks are hired to fill tanks that are also used to store rainwater. The borehole and rainwater quality appeared to be clear, but water treatment had not been performed, and the microbial quality was unknown. This shows that the Sustainable Development Goal (SGD) 6, clean water and sanitation, is still far from being met. According to national norms and standards for domestic water and sanitation services, people who do not use water treatment or purification techniques fall in the ‘no service’ category and contribute to the water backlog. Pit latrines (*n* = 46, 94%) and flush toilet (*n* = 3, 6%) were found to be the only convenient toilet systems used. The number of toilets is not sufficient according to the guidelines. There are (*n* = 46, 94%) of the schools in the study area using pit latrine due to insufficient or no water supply. In 89.8% of primary schools, sanitation facilities are in working condition in terms of repair and hygiene, while 10.2% are not usable in terms of hygiene, and these are mostly boy’s toilets. All schools (*n* = 46, 94%) that have flush toilets is because they received sponsorship from non-government stakeholders that funded them in achieving piped water systems that permit the functionality of flush toilets. For the purposes of this study, hygiene was evaluate based on the items found in toilets and handwashing practices. The hygiene aspects of toilets included tissues, cleanness, and toilet seat. For handwashing practices we looked the number of washing basins, the colour of water, and having soaps to use. In the schools that did provide handwashing facilities, some of the toilets were broken, there was no water, or there was no drainage system in place to allow them to function. However, according to the school act, the handwash basins should be inside the facilities. A total of (*n* = 7, 14%) of handwash basins were inside the toilets. Only (*n* = 2, 4%) of schools had handwashing facilities which were Jojo tanks with taps near toilets, which were outside of the toilet, with no soap provided. Additionally, (*n* = 40, 82%) of learners used drinking points for handwashing, which can possibly transmit microbes among them. The findings revealed that, in general, (*n* = 32, 64%) of school toilets were clean, while, in general, the girls’ toilets were cleaner than the boys’ toilets. In all the schools, the cleaning services were from the people who were involved in school nutrition. In conclusion, there were water sources available for access to water inside schools; however, the situation can be improved by increasing the number of water source points. Pit latrines were the main used toilets, which were in a majority of the schools, and did not have the necessary terms for hygiene such as handwashing basin, tissues, and others. The lack of the main aspect, i.e., access to water and sanitation items, results in an impact on hygiene to learners as they will fail to practice proper hygiene. However, improvement can still be made by keeping the boys’ toilets clean while increasing the number of handwashing basins inside the toilets, so that they do not use taps outside the toilets. Schools should work towards meeting the required number of handwashing basins to increase access to handwashing facilities.

## 1. Introduction

Water, sanitation, and hygiene (WASH) services are essential for human health and well-being. Moreover, WASH services are critical in providing a healthy and conducive learning environment, especially for children in primary schools [1]. Additionally, children are particularly vulnerable to WASH-related diseases, and access to adequate WASH services in schools is essential for their learning and development [2]. In many countries, including South Africa, primary schools start at grade R to grade 7 and accommodate learners from 6 to 12 years old. Children of these ages need a great deal of care and guidance in terms of handwashing, proper use of sanitation facilities, and general cleanliness. The state of WASH services in many primary schools around the world remains a concern. In this article, we will look at the current state of WASH services in primary schools and how it affects learners’ well-being and academic outcomes. Access to clean water is a fundamental right that is required for proper sanitation and hygiene practices. Unfortunately, many primary schools lack reliable and safe water sources. According to a report by the World Health Organisation (WHO) and the United Nations Children’s Fund (UNICEF), approximately 20% of schools worldwide do not have access to clean and safe drinking water [3]. A lack of adequate sanitation facilities in primary schools is another pressing issue. Proper toilets and handwashing stations are critical for encouraging good hygiene and preventing disease transmission. Surprisingly, approximately 50% of primary schools worldwide lack access to basic sanitation services, denying millions of children a safe and private place to relieve themselves [4]. The effects of inadequate WASH services in primary schools are far-reaching. For starters, a lack of access to clean water can cause learners to be absent more often [5].

Children whose only option to obtain drinking water is from unsafe water sources are more likely to contract waterborne diseases like diarrhoea and cholera, which can lead to repeated illnesses and missed school days [6,7]. Furthermore, without clean water, proper handwashing is difficult, increasing the risk of disease transmission. Inadequate sanitation facilities also have a negative impact on children’s health. The absence of proper toilets and hygiene facilities can lead to unsanitary practices, such as open defecation, which not only poses health risks but also compromises learners’ dignity and privacy [8]. Girls, in particular, may face additional challenges, such as a lack of menstrual hygiene management facilities, which can negatively affect their participation in school activities. Moreover, the poor state of WASH services in primary schools can hinder the overall educational experience of learners. Learners who lack access to clean water and sanitation facilities are more likely to feel uncomfortable and distracted in the classroom, impairing their ability to concentrate and learn effectively. This can contribute to lower academic performance and reduced educational outcomes. Addressing the status of WASH services in primary schools requires a multi-faceted approach [9].

Governments and policymakers should recognise the importance of investing in WASH infrastructure and allocate sufficient funds for its development and maintenance. Efforts should be made to provide clean and safe water sources within school premises, as well as establish appropriate sanitation facilities that meet the needs of all learners, including those with disabilities. Furthermore, promoting good hygiene practices should be an integral part of the primary school curriculum [10,11]. Teaching learners about handwashing techniques, proper sanitation, and menstrual hygiene management can go a long way in fostering healthy habits and preventing the spread of diseases [12]. Schools should also encourage the active participation of learners in maintaining a clean and hygienic environment by organising awareness campaigns and establishing student-led WASH clubs. In conclusion, the status of WASH services in primary schools remains a significant concern worldwide. The lack of access to clean water, sanitation facilities, and hygiene education has far-reaching consequences on the well-being and educational outcomes of learners. Governments, policymakers, and educational institutions must prioritise and invest in improving WASH infrastructure and practices in primary schools to ensure a healthy and conducive learning environment for all learners.

In South Africa, the provision of WASH services in schools is the responsibility of the Department of Education. However, there are significant challenges in meeting this responsibility, particularly in rural areas. In uMfolozi Local Municipality, Kwa-Zulu Natal, many primary schools still lack access to basic WASH services. What is not known is whether the primary schools in rural areas such as Kwa-Zulu Natal South Africa are properly supplying the WASH programme adequately. Therefore, this study aimed to evaluate the status of WASH in selected primary schools in the uMfolozi Local Municipality in the Kwa-Zulu Natal province. The specific objectives were to assess the availability and reliability of water supply that is used by learners in selected primary schools, to make an analysis of the status of sanitation and hygiene facilities in selected primary schools, and to draw conclusions and make recommendations on suitable ways to improve WASH services in selected primary schools.

## 2. Methods and Materials

### 2.1. Contextualisation of Study Area

**Figure 1:**
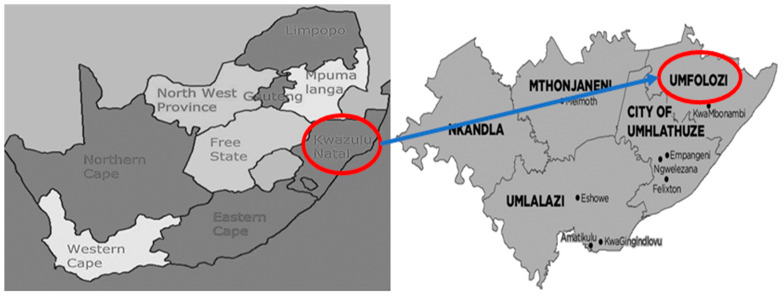
Map showing the location of Umfolozi (sourced from https://www.southafrica.to/provinces/provinces.php and https://municipalities.co.za/map/1106/umfolozi-local-municipality, accessed on 8 December 2024).

The study was conducted in uMfolozi Local Municipality in Kwa-Zulu Natal, South Africa. uMfolozi Local Municipality is one of the predominantly rural local municipalities in King Cetshwayo District Municipality, that have rural villages led by traditional leaders. uMfolozi Local Municipality has a population of 159,668 [13]. About 23.3% households have access to piped water inside the dwelling, and 26% of the households have access to flush toilets that are connected to sewerage system. The map showing the location of Umfolozi Local Municipality in the province of Kwa-Zulu Natal is presented in Figure 1.

### 2.2. Study Design

This study was a quantitative descriptive cross-sectional survey. Cross-sectional studies seek information from a sample at one point in time [14]. Traditionally, cross-sectional studies have been considered useful for determining the prevalence of a condition, hence they are also known as “prevalence studies” [15]. Cross-sectional studies do not follow individuals up over time; hence, they are usually inexpensive and easy to conduct. Moreover, they are useful for establishing preliminary evidence in planning a future advanced study [16].

The research was based on observations using a checklist to collect quantitative data from participating primary schools. This means that there was no intervention by the researcher in the outcome to be evaluated [15]. Observation as a data collection method is a mode of inquiry to systematically collect information about different settings and groups. The objective of observation in data collection is to better understand the phenomena of interest situated in context [17]. It provides the researcher with a unique and rare opportunity to collect first-hand information for looking, judging and interpreting things instead of relying on others [18]. Specifically, observation data collection can improve the understanding of practices, processes, knowledge, beliefs, and attitudes embedded in clinical work and social interactions [17].

### 2.3. Study Population and Sample Size

The study population was all the learners of the 60 primary schools that were in King Cetshwayo District municipality. The Raosoft^®^ sampling size calculation programme, 95% confidence level, 5% margin of error and 50% response distribution were used to determine the sample size. Purposive sampling method was used to select sample schools after obtaining a list of primary schools from King Cetshwayo District Municipality. The sample size was 53 schools, however; only 49 schools participated in the study.

### 2.4. Data Collection

Data were collected through observations using a checklist adapted from [19] to determine and assess the types and conditions of water supply sources and sanitation facilities, as well as hygiene habits and promotion. Specific aspects that were observed included WASH infrastructure, availability of essential items and equipment, and learners handwashing behaviour. All the three elements of WASH (i.e., water, sanitation and hygiene) were covered during observations.

### 2.5. Data Processing and Analysis

Data from the checklist were captured, cleaned and coded using MS Excel^®^ 2016, and thereafter were manually reviewed to ensure the validity of the data. The data were analysed using the Statistical Package for Social Science (SPSS version 29) (SPSS Inc., Chicago, IL, USA). The results are presented in the form of tables, pie charts and graphs.

Tables were used to group questions that had responses that were linked or most likely the same, e.g., questions that had yes/no responses. Additionally, pie charts were used to give a visual demonstration of the responses and enabled easy interpretation. Graphs were also used to give a visual demonstration of numerical responses.

### 2.6. Ethical Consideration

This study was approved by the Research Ethics Committee of Tshwane University of Technology (Ethics Clearance No. FCRE2022/05/013 (SCI) (FCPS 02)). Permission to carry out the study was obtained from Kwa-Zulu Natal Provincial Department of Education. As per approval letter instructions, copies of the letter were then submitted to King Cetshwayo District and Lower uMfolozi Circuit Offices, the Department of Education, and to the principals of the sampled primary schools.

## 3. Results

The results of this study is presented based on the analysis of the collected data regarding water, sanitation and hygiene (WASH) services in 49 primary schools in uMfolozi Local Municipality. The results are summarised in tables, pie charts and graphs and gives an overview of the provision and condition of drinking water, sanitation and hygiene facilities to ensure availability and accessibility of drinking water and sanitation services and promote good hygiene practices.

### 3.1. Descriptive Analysis of Learners in Schools

The descriptive analysis of learners in the schools that participated in the study is presented in Table 1 below. It shows the minimum and maximum number of learners, the average number of learners, the standard deviation and the 95% confidence interval, respectively.

It can be seen in Table 1 that there was a huge difference in the total number of learners per school, whereby the minimum number of learners was 60 and the maximum number was 1898. This is confirmed by the SD (216), which indicated a higher value, and therefore, more variability in deviation from the mean. It can be deduced from Table 1 that the distribution of the total number of learners per school was more unevenly spread out [20].

### 3.2. The Status of Water Availability and Reliability in Primary Schools

The types of drinking water supply sources used in schools are presented in Table 2 below.

Table 2 shows that the schools used three different drinking water supply sources. However, rainwater and schoolyard piped water (i.e., municipal water), were the most used drinking water supply sources (38.5%) in schools, with borehole being the least used source of water supply (23.1%). The observation revealed that the water from these sources were collected in either 5000 L or 10,000 L tanks, which were installed in schools. From the tanks, water was channelled to the drinking water points (i.e., taps) inside the schools. It can be deduced that the schools had variable sources of water supply to ensure availability of drinking water in schools, thus providing and enabling an environment for good hygiene. The number of drinking water points connected to each drinking water supply sources in schools is shown in Table 3 below.

It can be seen from Table 3 that rainwater points were the highest (156) followed by borehole and schoolyard piped drinking water points, respectively. It can also be seen that there was a slight difference in the number of drinking water points between borehole (31) and schoolyard piped (29) drinking water points.

The functionality of the water sources was also assessed by observing the functionality of the drinking water points connected to each water source. A water source is considered functional at a given moment in time if water is available from the source at that time; if water is not available (for any reason), the source is non-functional [21]. The results of functionality of the drinking water points connected to each water source are presented in Table 4.

Table 4 shows that drinking water service points connected to the rainwater supply system had a higher rate of functionality (76%) compared to the other sources of drinking water supply. Similarly, rainwater also had a higher rate of non-functionality than the other sources of water supply. This could be attributable to the fact that rainwater is collected when there is rainfall, which is a seasonal occurrence, thereby occasional. It could therefore be assumed that the rainwater storage tanks do run dry sometimes due to water usage and dry season, thus leading to the non-functionality of the connected drinking water service points. Table 4 further reveals an interesting observation, whereby the drinking water service points connected to the schoolyard piped water supply system had a higher rate of non-functionality (15%) compared to that of the borehole (9%), despite having a lower functionality rate (21%) compared to the borehole water service points’ functionality rate (26%). This could be attributed to the ability of the schools to promptly repair non-functional schoolyard piped water infrastructure due to factors such as access to tools and parts, services of a mechanic and availability of funds [21]. The results of the overall functionality (i.e., functionality index) of drinking water points connected to different drinking water sources in schools are presented in Table 5 below.

Table 5 shows that the overall functionality of the drinking water points connected to different water sources in schools was high (76%). This is encouraging given the fact that continuous service from safe, improved water sources is essential for maximising the benefits of safe water for human health and development [21]. Therefore, this high rate of functionality would promote good hygiene, particularly washing of hands by the learners in schools, thus preventing the spread of diseases. 

In Table 6, the safety of drinking water storage and water consumption areas was assessed. Majority of the schools had their tanks covered with lids to ensure that the water is not contaminated by external physical factors. Upon assessing the cleanliness of the area where water was being consumed in the schools that consumed water directly from standpipe taps, the consumption area was found to be clean and neat.

**Figure 2:**
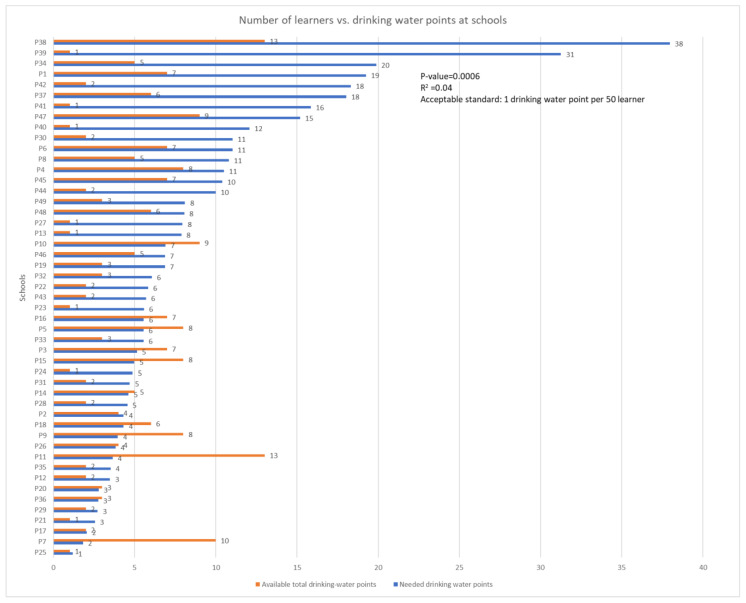
Number of available drinking water points vs. number of drinking water points needed in schools.

Figure 2 below presents the total number of available drinking water points versus the number of drinking water points needed in schools.

Figure 2 below shows the number of water points versus the number of learners in each school. Blue bars indicate the number of drinking water points that each school must have and orange bars indicate the number of drinking water points that were available in each school. By observing the graph below, a majority of the schools were non-compliant with the acceptable standard of drinking water points (1 drinking water point per 50 learners) [22], and some even surpassed the acceptable standard. This reflects poor planning by the Department of Education as the schools that were supposed to have more drinking water points as required by the standard had fewer and those that were required to have few had more. The relationship between the number of learners vs. drinking water points at the school was assessed using R-squared. The calculation of the R-squared was 0.04, meaning that only 4% of drinking water points are proportional to the number of learners in the school, while 97% of the drinking water points are not proportional to the number of learners in the school. Moreover, the *p*-value was 0.0006, which means that there is a statistically significant difference between the number of learners in school and the number of drinking points.

Accessibility to drinking water points was assessed for each school. Table 7 shows the frequency of the schools that had water-drinking points accessible to the smallest children at the school and quality characteristics for drinking water in each school. A total of (*n* = 46, 93%) of the 49 schools had water drinking points accessible to the learners at the school, and all (*n* = 49, 100%) schools recorded no colour, odour, taste and turbidity for water quality of the drinking water on the premises.

**Figure 3:**
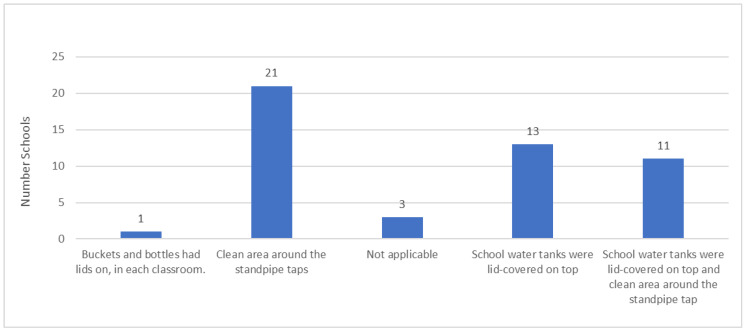
Measures to prevent drinking water contamination in schools.

Figure 3 below shows the measures taken at school to ensure that water is safe, clean and stored correctly in the schools. In the graph below, we can see that a majority of the school learners were drinking water directly from the standpipe that is from piped water into the schoolyard and that around those standpipes, it was clean enough to be a water drinking area. As for the schools that provided drinking water in buckets and bottles in classrooms, their containers had lids to protect water from contamination. For schools that consumed water from tanks located in classroom blocks, the tanks had plastic lids on top and only one school regularly treated water with Jik in school tanks. There was also one school that highlighted that they hire a cleaning company at intervals to clean inside the tanks and the gutter system that supplies water into the tanks. On the other hand, one school had no water supply at all, and instead, learners had to bring personal drinking bottles from their homes.

### 3.3. The Status of Sanitation Facilities in Schools

**Figure 4:**
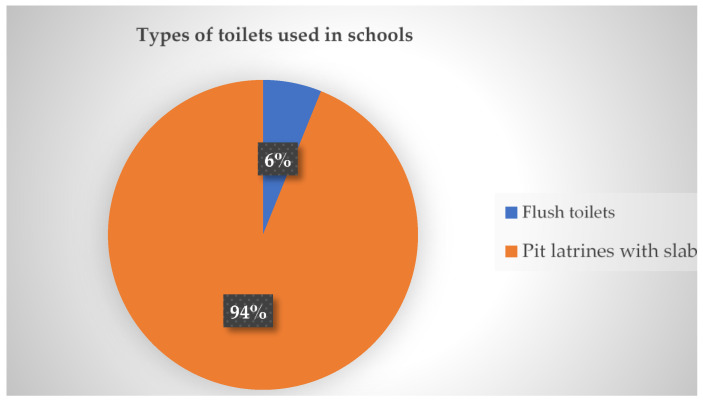
Types of toilets used in schools.

This section presents the results of the types of toilets in schools and they are presented in Figure 4 below.

It can be seen in Figure 4 that two types of toilet facilities were used in schools, namely flush and pit toilets. The results further show that pit toilets were used in (*n* = 46, 94%) schools, whereas flush toilets were used in (*n* = 3, 6%) schools.

**Figure 5:**
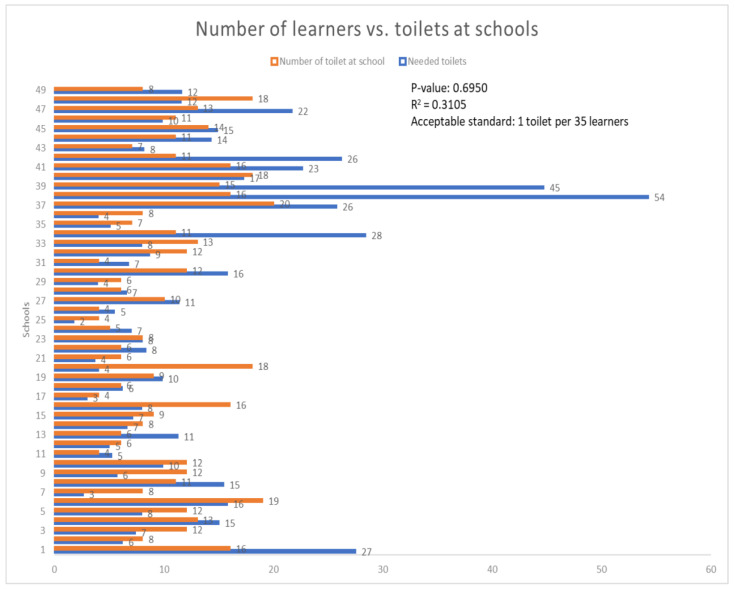
Number of toilets available vs. number of toilets needed in schools.

Figure 5 shows that all (*n* = 49, 100%) schools did have two or more toilet facilities for the learners. However, (*n* = 24, 48.98%) schools had sufficient toilet facilities (i.e., equal or more than the required number) in terms of the prescribed ratio of one toilet per thirty-five learners by UNICEF [23] In contrast, (*n* = 2, 51.02%) had an insufficient number of toilet facilities. It can therefore be deduced that all the schools that had sufficient toilet facilities promoted good sanitation and hygiene because learners had access to handwash basins/handwashing points, whereas those with insufficient toilet facilities constrained the promotion of good sanitation and hygiene, and thus exposed learners to potential outbreak of communicable diseases.

It can be seen in Figure 5 that the R^2^ pertaining to the total number of available toilets versus the total number of toilets needed was 0.3, which means that only 30% of the toilets were proportional to the number of learners in schools, while 70% of the toilets were not proportional to the number of learners in schools. This situation presented a risk of disease outbreak due to a lack of access to sanitation facilities in schools, which could potentially deprive learners of their dignity and good health.

### 3.4. The Status of Hygiene in Schools

This section presents the results pertaining to hygiene promotion and practices with a specific focus on posters or information promoting hygienic use of toilets or latrines and handwashing, provision of hot running water at handwashing facilities, and assessment of accessibility of handwashing facilities to learners, including those with limited mobility in schools. The results of hygiene promotion and practices are presented in Table 8 below.

Table 8 shows that (*n* = 48, 98%) schools did not have hot running water at their handwashing facilities and only one (2%) school had hot running water at its handwashing facilities. Additionally, handwashing facilities were accessible to all learners in (*n* = 45, 91.9%) schools. In the remaining four (8.1%) schools, some learners could not reach the handwashing facilities due to height. Moreover, handwashing facilities were not accessible to all learners with limited mobility (i.e., learners with disability) in all the (*n* = 49, 100%) schools.

**Figure 6:**
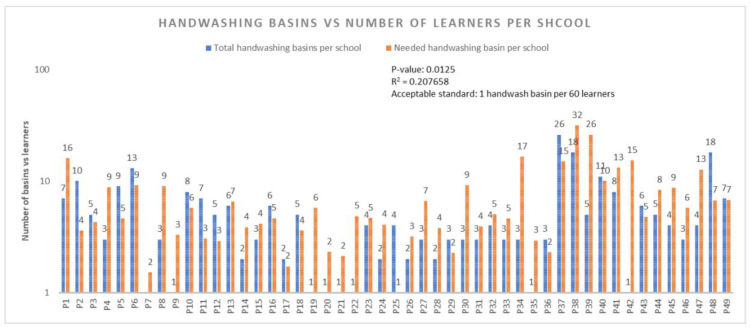
Available handwash basins/handwashing points vs. handwash basins/handwashing points needed in schools.

This suggests that when the handwashing facilities were constructed, height and disability of the learners were not taken into consideration, and therefore it presented problems to shorter learners and learners with disability, thus exposing them to the risk of contracting diseases due to not being able to wash their hands, especially after using the toilet. Table 8 further reveals that posters or information promoting hygienic use of toilets or latrines and handwashing were not observed in (*n* = 47, 95.9%) schools. Figure 6 below presents the results of available handwash basins/handwashing points versus handwash basins/handwashing points needed in schools.

Figure 6 shows that there is a significant difference between the total number of available handwash basins/handwashing points and the number of handwash basins/handwashing points needed in schools. Furthermore, (*n* = 48, 97.96%) schools did have one or more handwash basins/handwashing points and only one school did not have a handwash basin/handwashing point. Additionally, (*n* = 18, 36.73%) schools had sufficient handwash basins/handwashing points (i.e., more than the required number) in terms of the prescribed ratio of one handwash basins/handwashing points per sixty learners by the Department of Basic Education [22]. However, (*n* = 31, 63.27%) had an insufficient number of handwash basins/handwashing points. It can therefore be deduced that all the schools that had sufficient handwash basins/handwashing points promoted good hygiene because learners had access to handwash basins/handwashing points, whereas those with insufficient handwash basins/handwashing points constrained the promotion of good hygiene, thus exposing learners to potential outbreaks of communicable diseases.

It can be seen from Figure 6 that the R^2^ pertaining to the total number of available handwash basins/handwashing points versus the total number of handwash basins/handwashing points needed was 0.2, which means that only 20% of the handwash basins/handwashing points were proportional to the number of learners in schools, while 80% of the handwash basins/handwashing points were not proportional to the number of learners in schools. This situation presented a risk of the spread of diseases due to lack of access to handwashing facilities in schools, which could have promoted a culture of learners not washing their hands in schools.

## 4. Discussion

### 4.1. Water

The results of this study support evidence on WASH in schools in other nations and highlight typical WASH issues in schools. Sanitation and hygiene coverage in schools was substantially lower than water coverage, according to data analysis, and circumstances are probably worse than what was shown in the survey results. For example, almost all schools had water provision but none of them had a water quality-monitoring plan in place, which means that the quality of water consumed by learners is unknown in (*n* = 25, 36%) of schools. The rainwater that is stored in uncleaned tanks for months are at risk of continuous water quality contamination. While the water may not have shown any poor-quality characteristics during the survey, the risk is still there. The water is made available for various applications, such as human consumption, industrial purposes, or ecosystem preservation and must meet the quality requirements pertinent to those uses [24].

According to national norms and standards for domestic water and sanitation services, people who do not have access to safe water from an improved source fall in the ‘‘no service’’ category and contribute to the water backlog. These people typically obtain contaminated and unfit-for-drinking water from far-off rivers, springs, and/or tiny catchment dams and ponds. They do not use water treatment or purification techniques. To meet the Sustainable Development Goal (SDG) 6, clean water and sanitation, service providers must prioritise serving this segment of the population, and by 2030, everyone in South Africa must have access to safe water from an improved source. According to the Department of Water and Sanitation on water access and water treatment, 58% (rainwater and borehole) of primary schools in this study fall under the “no service” category and form part of a water backlog. As there are no proper or reliable water treatment or purification methods in any of the surveyed schools, this means that learners consume water from unimproved sources, and that is not in line with the 2030 SDGs regarding access to safe water [24].

The SDGs further state that consumers should be educated about water quality and its importance for human health, but on the other hand, none of the schools had visible educational information in place regarding drinking water [24]. The majority of the schools provide drinking water through 20 litres of buckets or bottles for their classrooms, whereas a volume of 15 to 20 litres of potable water per learner per day is the recommended quantity [24]. Even though there were no learners living with mobility disability in any of the studied schools, only one school was designed to have water points accessible to learners living with mobility disability. We do not know if the reason behind having no disabled learners in any of the schools is because of how all these schools are layered out or designed. In some schools where water is consumed straight from the tap or tank in the yard, the yard does not permit free movement of the wheelchair.

### 4.2. Sanitation

One of the biggest issues we found concerning sanitation facilities was that (*n* = 19, 45%) of the schools do not have toilets designed for the smallest children; instead, they share with the older learners. It was stated that class teachers accompany them, but in one school, a learner was observed to have difficulty climbing the toilet seat with his hands. A study on the critical analysis of the school pit toilet system revealed that there are numerous horrifying accounts of inadequate sanitary facilities in schools, particularly those in rural areas, which occasionally lead to student fatalities. Numerous learners have been reported missing from schools and found in pit restrooms close to the classrooms a few hours after being missing [25]. This poses a very serious health concern, especially taking into consideration that none of these schools are providing soap for thorough handwashing. Another great concern was that none of the schools had a private disabled toilet available for functionality, and the schools that had them were either used by teachers and other learners or used as a storage. According to [26], learners with disabilities who lack access to sanitary facilities often become frustrated and act in ways that cause stigma and discrimination from those around them. One school had a toilet building with no ventilation system besides windows, which was against the National Building Regulations and Building Standards Act (Act 103 of 1977) that outlines acceptable ventilation requirements. This resulted in the toilet facility having a strong odour with flies and learners avoiding their use. In about five schools, the teachers do not have their own toilets but have to share with the learners, which seemed to be a very unpleasant situation.

### 4.3. Hygiene

If taps are utilised, they must be supplied straight from the main source and marked as drinkable. Sinks need to be clean and empty, and the area needs to be hygienic [27]. During our survey, we found learners drinking straight from the main tap and only one school provided metal drinking cups that were being shared, and there was no clear indication as to by whom, how and when the cups are hygienically cleaned. The very same taps used for drinking were also used for handwashing in the majority of the schools, which poses a hygienic concern. Where water supply and sanitation facilities are insufficient or non-existent, the effectiveness of hygiene promotion in schools as well as good hygiene behaviour is severely constrained. Teachers cannot effectively promote proper toilet usage if they avoid using them because the facilities are unclean or hazardous, or express the value of handwashing if there is no water or soap available in the toilets [28]. Likewise, some schools seemed to have given up on good hygiene and sanitation practices because of no provision of relevant resources. Schools should run WASH advocacy programmes to educate learners on the value of being hydrated and the role that water plays in human health, as well as to actively encourage water intake during breakfast, during breaks, and throughout lunch [27]. In addition, none of the schools had WASH educational information on display.

## 5. Conclusions

An evaluation of the status of WASH services led to the conclusion that the status of WASH services is very poor or not in a good state in the study area. Almost all schools had water available at the school but those that were dependent on rainwater were consuming unsafe water from the school tanks. Since there was no water treatment plan in place, even those who claimed that they do treat their water supplies, their responses could not be relied on because there was no evidence. The other major water-related issue is that most schools use water drinking points as washing points as well, which can result in water source contamination or cross-contamination. The lack of maintenance for sanitation and hygiene facilities indicated that school administrators have been ignorant about the significance of the utilisation of safe and hygienic facilities.

## 6. Recommendations

The following recommendations are made to improve the status of WASH services in uMfolozi primary schools:

- Increase investment in WASH infrastructure: The government should increase investment in WASH infrastructure in uMfolozi primary schools. This includes providing schools with access to reliable sources of clean water, constructing adequate sanitation facilities, and installing hygiene facilities.
- Strengthen WASH management and capacity building: The government should strengthen WASH management and capacity building at the school level. This includes training school staff on WASH management and providing them with the resources they need to implement effective WASH programmes.
- Promote community participation in WASH: The government should promote community participation in WASH. This includes working with communities to identify WASH needs, develop WASH plans, and implement WASH programmes. A conference paper by [29] revealed that in the provinces of Bam and Sanmatenga in Burkina Faso, three community self-assessments are conducted each year; at the start, middle, and end of the academic year. In order to review indicators for each target and ascertain the degree of success and difficulties, the WASH committee conducts direct observations of the school’s water, sanitation, and hygiene facilities as well as monitoring visits that involve interactive interactions between the various actors of the school community.

By implementing these recommendations, the government can improve the status of WASH services in uMfolozi primary schools and create a healthier and more conducive learning environment for all children. A study by [30] conducted in India also stated that both internal and external environments contain physical barriers. Parents and children with disabilities may encounter difficulties travelling to and from school. Barriers in the external environment, such as paths from home to school, safe and accessible roads, crossings, and accessible transportation, must be addressed. To guarantee that children with disabilities can participate in education, it is also essential to make sure that all school facilities are accessible to them.

## 7. Limitations

We could not confirm or have confidence that the rainwater from school tanks and water from boreholes was adequately safe and clean for human consumption since no samples were taken; additionally, there was no treatment process in place in any of the affected schools.

## Figures and Tables

**Table 1:** Summary descriptive analysis of learners from 49 participated schools.

	Count	Mean	Std Deviation	Min	Max	Con. Level 95%
Number of learners	49	424	216	60	1898	104

**Table 2:** Types of drinking water sources used in schools.

Types of Drinking Water Sources	Frequency	%
Borehole	15	23.1%
Rainwater	25	38.5%
Schoolyard piped water	25	38.5%
Total	65	100%

**Table 3:** Number of drinking water points connected to each drinking water supply sources in schools.

Number of Drinking Water Points	Frequency	%
Borehole	31	14%
Rainwater	156	72%
Schoolyard piped water	29	13%
Total	216	100%

**Table 4:** Functionality of drinking water points by type of water source in schools.

No. of Functional Drinking Water Points	N	%	No. of Non-Functional Drinking Water Points	N	%
Borehole water	26	16	Borehole water	5	9
Rainwater	116	71	Rainwater	40	76
Schoolyard piped water	21	13	Schoolyard piped water	8	15
Total	163	100	Total	53	100

**Table 5:** Functionality index of drinking water points in schools.

	N	%
Functioning drinking water points	163	76%
Non-functioning drinking water points	53	24%
Total	216	100%

**Table 6:** Safety of drinking water storage tanks in schools.

Safety Aspect	N	%
Water tank covered with tight-fitting lid	26	43%
Area around the standpipe tap	32	53%
Water storage tank treated regularly with Jik	2	3%
Total	70	100%

**Table 7:** Accessibility of drinking water points by learners in schools.

Accessibility to Drinking Water Points	N (Yes)	Percentage (%)
Is at least one drinking water point accessible to the learners at the school?	46	93%
What quality characteristics apply to the drinking water on the premises? Water has no colour, odour, taste and no turbidity	49	100%

**Table 8:** Hygiene promotion and practices.

	N (Yes and %)	N (No and %)
Are there any posters promoting hygienic use of the toilets?	2 (4.1%)	47 (95.9)
Is running hot water provided at handwashing facilities?	1 (2.0%)	48 (98%)
Are handwashing facilities accessible to those with limited mobility?	0 (0%)	49 (100%)
Are handwashing facilities accessible to the learners in schools?	45 (91.9%)	4 (8.1)
Is any information about handwashing/hand hygiene visible at the school?	2 (4.1%)	47 (95.9%)

## Data Availability

The data presented in this study are available on request from the corresponding author due to privacy and ethical reasons.
